# Unusual Presentation of Acute Gastrointestinal Bleeding in Gastric Lipoma and Concomitant *Helicobacter pylori* Infection: A Case Report

**DOI:** 10.1002/ccr3.70311

**Published:** 2025-03-14

**Authors:** Amey Joshi, Rohan Kumar, Maitri Shah, Ryan Mui, Tadd Kaeo Hiatt

**Affiliations:** ^1^ Internal Medicine Michigan State University Lansing Michigan USA; ^2^ Department of Gastroenterology, Clinical Faculty Michigan State University Lansing Michigan USA; ^3^ Department of Gastroenterology University of Michigan Ann Arbor Michigan USA

**Keywords:** gastric lipoma, GI bleed, *helicobacter pylori*, stool antigen testing

## Abstract

Active *Helicobacter pylori* ( *H. pylori* ) infection may contribute to the ulceration and hemorrhage of otherwise benign gastric lipomas. This case highlights the utility of combining invasive and non‐invasive testing of *H. pylori* in bleeding gastric ulcers and emphasizes the critical role of *H. pylori* testing and eradication in bleeding gastric lipomas.

## 1 Introduction

Gastric lipomas are rare tumors accounting for < 1% of all gastric neoplasms and 5% of all gastrointestinal lipomas [1]. Gastric lipomas are usually discovered incidentally on imaging or endoscopy and managed conservatively, as the majority remain asymptomatic. Complications of gastric lipomas are rare and usually occur for lesions > 2 cm and include bleeding, gastric outlet obstruction, and gastroduodenal intussusception [2, 3].

*Helicobacter pylori* ( *H. pylori* ) infection is associated with peptic ulcer disease and ulcer‐related bleeding, and eradication improves outcomes, including re‐bleeding events and the need for blood transfusions. It is unclear if *H. pylori* infection increases the risk of gastric lipoma‐related bleeding. Here, we present a rare case of life‐threatening bleeding from an ulcerated gastric lipoma in the setting of *H. pylori* infection. This case underscores the importance of *H. pylori* testing and eradication in the management of ulcerated gastric lipomas.

## 2 Case Presentation

A 69‐year‐old male with a past medical history of known gastric lipoma, Barrett's esophagus, type 2 diabetes mellitus, chronic kidney disease stage 4, and coronary artery disease presented to the emergency department with complaints of worsening exertional dyspnea, generalized weakness, post‐prandial periumbilical pain, and black tarry stools. He denied any recent use of non‐steroidal anti‐inflammatory drugs, tobacco, alcohol, or illicit drugs.

## 3 Investigations and Treatment

**FIGURE 1:**
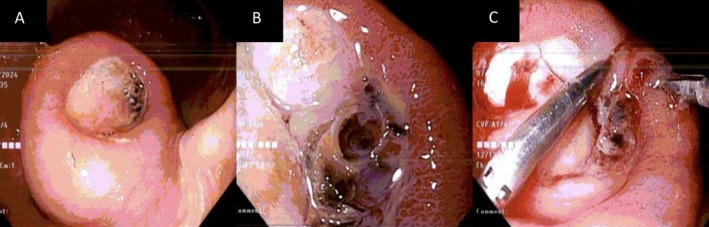
Esophagogastroduodenoscopy showing (A) 4 × 4 cm mass in the gastric antrum, (B) ulceration over the mass visualized with visible vessel, and (C) visible vessel was hemoclipped.

Laboratory investigations revealed severe acute anemia, with a hemoglobin of 6.1 g/dL, leukocytosis (15.1/mm^3^), elevated serum creatinine of 3 mg/dL (baseline 2.8 mg/dL), and uremia (blood urea nitrogen of 137 mg/dL). He was started on intravenous pantoprazole and resuscitated with blood transfusions and intravenous fluids. Due to concerns of brisk upper gastrointestinal bleeding, he was taken for urgent esophagogastroduodenoscopy (EGD) that revealed large amounts of old blood obscuring an atypically appearing 4 × 4 cm edematous mass with a large adherent clot in the distal gastric corpus. Irrigation was attempted to unroof the clot; however, this was not successful despite multiple attempts. No active bleeding was observed, and intervention was not pursued. The patient subsequently had two large episodes of melena, and his hemoglobin dropped from 7.2 to 5.7 g/dL. He was taken for a repeat EGD, revealing that the previously seen mass had a large ulcerated center containing a visible vessel (Figure 1A,B). The mass was biopsied for histopathology due to initial concerns for stromal tumors. Local epinephrine was then injected, and the vessel was hemoclipped (Figure 1C).

## 4 Results and Conclusion

The patient's hemoglobin remained stable throughout his admission, and he was discharged with oral pantoprazole. On subsequent follow‐up, the pathology of the lesion was consistent with a lipoma. *H. pylori* stool antigen was positive. To eradicate Hp, he was treated with pantoprazole, bismuth subsalicylate, metronidazole, and tetracycline for 14 days. On repeat laboratory testing, the patient's hemoglobin was stable, and he denied any episodes of melena, frank bloody stools, or hematemesis.

Although gastric lipomas rarely present with complications, active *H. pylori* infection may predispose these otherwise benign lesions to ulcerate and cause life‐threatening hemorrhage. The present case also highlights the importance of combining non‐invasive and invasive testing in bleeding gastric ulcers to yield the best diagnostic accuracy. The choice of diagnostic tests should be based on clinical context, patient preference, and resource availability.

## 5 Discussion

Gastric lipomas are mesenchymal tumors rarely associated with life‐threatening bleeding. Although the size of gastric lipomas (> 2 cm) has been associated with gastric outlet obstruction, intussusception, and bleeding, concurrent *H. pylori* infection has rarely been documented as a potential risk factor for severe bleeding in these tumors [4, 5]. The present case highlights the possibility that active *H. pylori* infection may increase the risk of bleeding in the otherwise benign course of gastric lipomas.

The pathophysiology of *H. pylori* infection is complex and differs greatly between acute and chronic stages. In acute infections, *H. pylori* produces urease to hydrolyze urea to ammonia, which neutralizes gastric acid production locally, creating a favorable environment for colonization. Ammonia, fatty acids, and cytokines, including interleukin 1β, produced by the bacterium, have an inhibitory effect on the H^+^/K^+^‐ATP‐ase pump, which reduces gastric acid production. The transition from the acute phase of *H. pylori* infection to chronic is accompanied by the restoration of gastric acid production. If colonized in the gastric antrum, the alkalinization caused by the locally produced ammonia increases gastrin release and, thereby, gastric acid production, predisposing the mucosa to breakdown and ulceration [6]. In the present case, the location of the gastric lipoma was also in the antrum of the stomach, which may explain why the *H. pylori* infection may have caused ulceration and subsequent bleeding.

Testing for *H. pylori* can be invasive (histopathological evidence) or non‐invasive (stool antigen test). Histopathological testing for *H. pylori* infection is generally more sensitive and specific than stool antigen testing, with rates as high as 95% and 98% compared to 92% and 95%, respectively [7, 8, 9]. However, the sensitivity of histopathology decreases in patients with acute peptic ulcer bleeding or those on proton pump inhibitor (PPI) therapy. Despite histology generally having higher sensitivity and specificity, our case yielded a negative result, with a positive stool antigen test. Visualization of the gastric antrum was limited due to the ongoing GI bleed, which may have limited the quality of the obtained biopsy samples, resulting in a false negative histology result. Meanwhile, the stool antigen monoclonal enzyme immunoassay remains sensitive even in an ongoing GI bleed, as seen in the present case.

Although most commonly benign, gastric lipomas should be treated or excised when symptomatic or are > 2 cm in size [10, 11]. Prior to the current admission, our patient was diagnosed with a gastric lipoma on diagnostic endoscopy for abdominal pain; however, as surveillance is not routinely recommended, this was not monitored. This warranted the question of whether intervention could be provided at the time of diagnosis itself, given the abdominal pain as the sole symptom. Furthermore, earlier intervention may have prevented the life‐threatening gastrointestinal hemorrhage with which the patient presented.

## Author Contributions

**Amey Joshi:** conceptualization, data curation, funding acquisition, investigation, methodology, project administration, resources, supervision, validation, writing – original draft, writing – review and editing. **Rohan Kumar:** investigation, methodology, project administration, writing – original draft, writing – review and editing. **Maitri Shah:** investigation, methodology, resources, writing – original draft, writing – review and editing. **Ryan Mui:** conceptualization, data curation, formal analysis, methodology, project administration, supervision, validation, writing – original draft, writing – review and editing. **Tadd Kaeo Hiatt:** formal analysis, funding acquisition, investigation, methodology, project administration, supervision, validation, visualization, writing – review and editing.

## Consent

Written informed consent was obtained from the patient to publish this report.

## Conflicts of Interest

The authors declare no conflicts of interest.

## Data Availability

The data supporting the findings of the present study are available from corresponding author upon request.

## References

[1] W. M. Thompson , A. I. Kende , and A. D. Levy , “Imaging Characteristics of Gastric Lipomas in 16 Adult and Pediatric Patients,” AJR. American Journal of Roentgenology 181, no. 4 (2003): 981–985. 14500213 10.2214/ajr.181.4.1810981

[2] S. A , U. DC , P. V. AA , H. N , R. R , and B. K , “A Rare Case of Gastric Lipoma Presenting With Gastric Outlet Obstruction Treated Endoscopically,” Case Reports in Gastrointestinal Medicine 2019 (2019): 5749830. 30906601 10.1155/2019/5749830 PMC6393926

[3] P. Kumar and C. Gray , “Gastric Lipoma: A Rare Cause of Gastrointestinal Bleeding,” ANZ Journal of Surgery 87, no. 9 (2017): 741–742. 25708232 10.1111/ans.13019

[4] M. Cherif , M. Mesbahi , N. Khedhiri , Y. Benzarti , and A. B. Maamer , “Gastric Lipoma: An Unusual Cause of the Upper Gastrointestinal Bleeding,” International Journal of Surgery Case Reports 119 (2024): 109684, 10.1016/j.ijscr.2024.109684. 38718494 PMC11091505

[5] M. E. Abdulrahman , A. Aji , and M. B. Alsabek , “Incidental Giant Obstructed Pedunculated Gastric Lipoma During Gastrostomy: A Case Report,” International Journal of Surgery Case Reports 53 (2018): 433–435, 10.1016/j.ijscr.2018.11.052. 30567062 PMC6262802

[6] H. L. Waldum , P. M. Kleveland , and Ø. F. Sørdal , “ *Helicobacter pylori* and Gastric Acid: An Intimate and Reciprocal Relationship,” Therapeutic Advances in Gastroenterology 9 (2016): 836–844, 10.1177/1756283X16663395. 27803738 PMC5076771

[7] B. Braden , G. Teuber , C. F. Dietrich , W. F. Caspary , and B. Lembcke , “Comparison of New Fecal Antigen Test With (13)C‐Urea Breath Test for Detecting *Helicobacter pylori* Infection and Monitoring Eradication Treatment: Prospective Clinical Evaluation,” BMJ 320 (2000): 148, 10.1136/bmj.320.7228.148. 10634733 PMC27260

[8] R. M. Genta and D. Y. Graham , “Comparison of Biopsy Sites for the Histopathologic Diagnosis of *Helicobacter pylori* : A Topographic Study of *H. pylori* Density and Distribution,” Gastrointestinal Endoscopy 40, no. 3 (1994): 342–345, 10.1016/s0016-5107(94)70067-2. 7794303

[9] D. O. Faigel , M. Childs , E. E. Furth , A. Alavi , and D. C. Metz , “New Noninvasive Tests for *Helicobacter pylori* Gastritis. Comparison With Tissue‐Based Gold Standard,” Digestive Diseases and Sciences 41, no. 4 (1996): 740–748, 10.1007/BF02213130. 8674395

[10] S. Termos , O. Reslan , O. Alqabandi , et al., “Giant Gastric Lipoma Presenting as GI Bleed: Enucleation or Resection?,” International Journal of Surgery Case Reports 41 (2017): 39–42, 10.1016/j.ijscr.2017.10.004. 29031177 PMC5645482

[11] A. Caliskan , W. K. Kohlmann , K. E. Affolter , E. Downs‐Kelly , P. Kanth , and M. P. Bronner , “Intramucosal Lipomas of the Colon Implicate Cowden Syndrome,” Modern Pathology 31, no. 4 (2018): 643–651, 10.1038/modpathol.2017.161. 29192650

